# Integrated Transcriptomics and Metabolomics Analysis Reveals *IbCCoAOMT7* Negatively Regulating Anthocyanin Accumulation in Sweetpotato Storage Roots

**DOI:** 10.3390/biology15141102

**Published:** 2026-07-08

**Authors:** Xinliang Liu, Tianqi Gao, Meng Kou, Mengjiao Lan, Zhiyuan Gao, Chen Li, Xinru Yu, Zongyun Li, Qiang Li

**Affiliations:** 1 School of Life Sciences, Jiangsu Normal University, Xuzhou 221116, China; liuxinliang0558@sina.com (X.L.); lanmj100@163.com (M.L.); gaozhiyuan2025@126.com (Z.G.); zongyunli@jsnu.edu.cn (Z.L.); 2 Xuzhou Institute of Agricultural Sciences in Jiangsu Xuhuai District/Key Laboratory of Biology and Genetic Breeding of Sweetpotato, Ministry of Agriculture and Rural Affairs, Xuzhou 221131, China; 20230062@jaas.ac.cn (T.G.); lichen@jaas.ac.cn (C.L.); 3 Fuyang Academy of Agricultural Sciences, Fuyang 236065, China; 2018101097@njau.edu.cn

**Keywords:** Sweetpotato (*Ipomoea batatas* (L.) Lam.), secondary metabolism, carbon flux allocation, phenylpropanoid pathway, anthocyanin biosynthesis, lignin biosynthesis, *Metabolic regulation*

## Abstract

Anthocyanins dictate the nutritional quality of purple-fleshed sweetpotato (PFSP), yet how the lignin pathway influences anthocyanin biosynthesis and phenylpropanoid metabolic flux allocation remains largely elusive. Through integrated transcriptomic and metabolomic analyses of a purple-fleshed sweetpotato cultivar (XZ13) and its natural mutant (XZ13M), we revealed that diminished anthocyanin accumulation correlated strongly with systemic transcriptional repression of the phenylpropanoid pathway. We subsequently identified a crucial methyltransferase gene, *IbCCoAOMT7*, with dual nucleocytoplasmic localization. Its overexpression in its storage roots was directly associated with reduced anthocyanin accumulation and increased lignin content. Further investigations suggested that *IbCCoAOMT7* was putatively involved in modulating metabolic flux toward lignin biosynthesis that downregulates the transcriptional activity of core anthocyanin biosynthetic genes. Collectively, our study highlights that *IbCCoAOMT7* represents a promising candidate gene that influences the balance between the lignin and anthocyanin pathways. These findings propose a tentative regulatory model, possibly involving substrate availability and transcriptional feedback inhibition, providing precise genetic targets for breeding anthocyanin-enriched sweetpotato.

## Simple Summary

Purple-fleshed sweetpotatoes derive their nutritional benefit from anthocyanins, which compete with lignin for plant carbon resources. Through a comprehensive analysis of the natural mutant XZ13 and subsequent experimental validation, we identified *IbCCoAOMT7* (Caffeoyl-CoA-O-methyltransferase) as a candidate gene associated with this metabolic balance. This study demonstrated that the expression of *IbCCoAOMT7* correlates with a reduction in anthocyanin accumulation and a concurrent increase in lignin content. Consequently, we conservatively propose that *IbCCoAOMT7* might influence this allocation trade off, suggesting a potential genetic target for breeding nutritionally enhanced varieties.

## 1. Introduction

Sweetpotato (*Ipomoea batatas* (L.) Lam.) is an essential global food crop and industrial raw material [1]. In recent years, purple-fleshed sweetpotato (PFSP) has attracted extensive attention from breeders and consumers due to their vibrant coloration and remarkable health-promoting functions, which are primarily attributed to the massive accumulation of anthocyanin in their storage roots [2]. Sweetpotato anthocyanins not only exhibit potent in vitro and in vivo antioxidant activities but also effectively reduce the risks of cardiovascular diseases and certain metabolic syndromes [3]. Therefore, comprehensively elucidating the biosynthetic and metabolic regulatory networks of anthocyanin in sweetpotato storage roots holds profound significance for cultivating novel sweetpotato varieties with high quality and added value via molecular breeding strategies.

Anthocyanin biosynthesis belongs to the massive phenylpropanoid pathway within plant secondary metabolism. Serving as a highly energy-consuming metabolic network and a tremendous carbon sink in plants, this pathway is catalyzed by a cascade of structural genes, including *PAL*, *CHS*, and *DFR*, and is delicately orchestrated at the transcriptional level by the spatiotemporal network of the MBW protein complex [4]. Notably, while supplying precursors for anthocyanin, the phenylpropanoid pathway is concurrently the fundamental source for lignin biosynthesis. These two metabolic branches share critical substrates such as p-coumaroyl CoA and caffeoyl-CoA during their initial steps. This implies that under the restricted carbon allocation system in plants, an innate metabolic competition and antagonistic relationship exists between anthocyanin and lignin biosynthesis.

Mounting evidence confirms that artificial intervention at key nodes of the lignin biosynthetic pathway can significantly alter anthocyanin accumulation patterns by modulating metabolic flux. When the lignin synthesis pathway is obstructed, the upstream accumulated carbon precursors are frequently reallocated to the flavonoid branch, thereby promoting anthocyanin biosynthesis [5]. For instance, the functional mutation of *ZmGCH1* not only significantly attenuated cell wall lignin deposition but also successfully drove the reallocation of carbon precursors toward the flavonoid branch, initiating substantial anthocyanin accumulation [6]. In Zijuan tea (*Camellia sinensis*), the natural downregulation of *CCR1* and related peroxidases effectively restricted the flow of intermediate metabolites toward lignin polymers and forcefully redirected the metabolic flux into the anthocyanin biosynthetic pathway, significantly intensifying the purplish-red coloration of the leaves [7]. Furthermore, hydroxycinnamoyl transferase serves as a core rate-limiting enzyme controlling the entry of substrates into lignin-specific monomer synthesis, and its expression level strictly governs the destination of carbon flux. Studies demonstrated that in *Medicago sativa*, specifically knocking down *HCT* expression via RNA interference fundamentally blocked substrate consumption directed toward lignin, which subsequently reactivated and amplified the metabolic flux of the flavonoid and anthocyanin pathways, inducing a massive accumulation of defensive pigments [8]. In summary, genes facilitating lignin biosynthesis likely act as competitors or negative regulators of anthocyanin accumulation within the metabolic network. However, the relationship between these two branches is not exclusively antagonistic. Previous studies have also described alternative regulatory mechanisms where both pathways are synergistically co-activated by upstream transcription factors under environmental stress or balanced through complex transcriptional feedback networks.

In the specialized underground organ of sweetpotato storage roots, it remains unclear whether lignin pathway genes influence anthocyanin accumulation through analogous metabolic competition mechanisms. Using purple-fleshed sweetpotato and their natural mutants as materials, this study mined and identified *IbCCoAOMT7*, a key lignin synthesis gene significantly associated with anthocyanin accumulation, via integrated transcriptomic and metabolomic analyses. We hypothesized that *IbCCoAOMT7* acts as a metabolic sink in sweetpotato storage roots, where its robust expression negatively regulates anthocyanin biosynthesis by redirecting shared carbon precursors toward the lignin branch. To test this hypothesis, we utilized a transgenic system to evaluate whether *IbCCoAOMT7* could directly influence anthocyanin and lignin accumulation. This study confirmed that the overexpression of *IbCCoAOMT7* significantly suppressed anthocyanin accumulation and promoted lignin deposition. Consequently, we propose a putative competitive model, wherein IbCCoAOMT7 may negatively regulate anthocyanin biosynthesis by competing for shared substrates and redirecting metabolic flux toward the lignin branch. These findings provide a novel theoretical basis and genetic targets for the molecular breeding of high-anthocyanin-content sweetpotato.

## 2. Materials and Methods

### 2.1. Plant Materials and Treatments

The sweetpotato varieties utilized in this study were XuZi13 (XZ13, characterized by purple skin and purple flesh) and its natural mutant (XZ13M, characterized by red skin and yellowish flesh). Multi-year and multi-location evaluations confirmed the stable inheritance of their traits from 2023 to 2025. The experimental materials were cultivated in the sweetpotato breeding experimental field of the Xuzhou Institute of Agricultural Sciences in Jiangsu Xuhuai District. At each location, a randomized complete block design was employed with three replicates and 20 plants per plot. To minimize environmental variance during downstream molecular evaluations, specific samples were exclusively collected from the Xuzhou location during the 2025 growing season. Mature sweetpotato storage roots were harvested 120 days post-planting. Sample selection strictly required uniform size and a complete absence of physical damage or visual disease symptoms. To generate robust biological replicates, tissue samples were pooled from three independent plants randomly chosen from distinct plots. This specific pooling strategy established three independent biological replicates utilized across all experimental analyses. Subsequently, the samples were transferred to an ultralow temperature freezer at −80 °C for future use. Three biological replicates were included in all experimental analyses.

### 2.2. Determination of Total Anthocyanin Content in Storage Roots

The extraction and determination of total anthocyanin content in the storage roots were performed according to the method described by Lan [9]. The brief procedures are as follows. A 400 mg aliquot of fresh tissue was mixed with 10 mL of a methanol and hydrochloric acid solution (99 to 1, *v*/*v*) and extracted by shaking in the dark for 18 h. Following high speed centrifugation, the supernatant was diluted 10-fold with the extraction buffer. The absorbance was measured at wavelengths of 530 nm and 657 nm using a standard 1 cm path length cuvette. The pure extraction buffer was used for blank correction. Relative anthocyanin content was calculated according to the formula: (A530 − 0.25 × A657)/g fresh weight, and expressed as relative units [10].

### 2.3. Targeted Anthocyanin Metabolomic Analysis of Storage Roots

Lyophilized samples were ground in liquid nitrogen, and 50 mg of the resulting powder was dissolved in 1.2 mL of 70% methanol precooled to −20 °C. The mixture was subjected to intermittent vortexing for 30 s every 30 min for a total of 6 cycles and then centrifuged at 12,000 rpm for 3 min. The supernatant was filtered through a 0.22 µm membrane. To monitor the stability and repeatability of the analytical instrument, quality control samples were prepared by pooling equal aliquots from all individual experimental samples and were injected at regular intervals throughout the analytical sequence. Targeted metabolomic analysis was commissioned to Wuhan Metware Biotechnology (Wuhan, China) and executed on ultra performance liquid chromatography tandem mass spectrometry platform (ExionLC AD tandem QTRAP 6500 plus, SCIEX, Marlborough, MA, USA). Chromatographic separation utilized an Agilent SB-C18 column (1.8 µm, 2.1 mm × 100 mm; 40 °C) with a mobile phase gradient of 0.1% formic acid in water (A) and acetonitrile (B). Data were acquired in multiple reaction monitoring (MRM) mode utilizing an ESI source (500 °C; curtain gas: 35 psi; ion spray: 5.5 kV/−4.5 kV) with metabolite-optimized declustering potentials and collision energies. In MRM mode, the first quadrupole screened target precursor ions to eliminate background interference, followed by collision-induced dissociation and subsequent fragment ion selection in the triple quadrupole to optimize quantification precision. Metabolites were annotated via the proprietary MWDB database 2023 (Metware Biotechnology Co., Ltd., Wuhan, China) and quantified using Analyst 1.6.3 software (Sciex). For absolute quantification of core anthocyanin derivatives, authentic standard solutions were prepared at serial concentrations of 0.01, 0.05, 0.1, 0.5, 1, 5, 10, 50, 100, 500, 1000, 2000, and 5000 ng/mL to construct linear calibration curves (Table S6). The absolute metabolite content was calculated using the following formula: Content (μg/g) = (c × V)/(1,000,000/m). Quality control (QC) pools monitored instrument stability, and multivariate analyses (PCA, OPLS-DA) were executed in R v4.5.2. Differentially accumulated metabolites (DAMs) were filtered based on false discovery rate (FDR < 0.05). Strict criteria (VIP ≥ 1, |log_2_FC| ≥ 1) were applied to sweetpotato comparisons (‘XZ13’ vs. ‘XZ13M’) [11].

### 2.4. Extraction of Total RNA and DNA from Storage Roots and Gene Sequence Amplification

Total RNA extraction from all storage roots involved in this study was performed using a plant RNA extraction kit designed for polysaccharide- and polyphenolic-rich tissues (Huayueyang Biotechnology, Beijing, China). After verifying the RNA purity and integrity, cDNA was synthesized by reverse transcription using the ReverTra Ace qPCR RT Master Mix with gDNA Remover kit (TOYOBO Life Science, Osaka, Japan). Using the XZ8 cDNA as a template, specific primers (primer sequences are detailed in Table S4) and PrimeSTAR Max high fidelity DNA polymerase (Takara Bio Inc., Kusatsu, Shiga, Japan) were employed to amplify the coding sequences of target genes. All procedures were strictly executed according to the manufacturer instructions. Genomic DNA extraction was conducted using the traditional cetyltrimethylammonium bromide method [12].

### 2.5. Transcriptomic Sequencing Analysis of Storage Roots

Polyadenylated mRNA-enriched libraries were constructed using the NEBNext Ultra RNA Library Prep Kit (New England Biolabs, Ipswich, MA, USA) and sequenced on an Illumina HiSeq platform, generating 150 bp paired end reads. Clean reads were obtained by removing adapters and filtering out low quality reads, which were subsequently mapped to the *Ipomoea batatas* Beauregard v4.0 reference genome utilizing HISAT2 software v2.2.1 [13]. Gene-level unnormalized raw read counts were generated using featureCounts software v2.0.3 and strictly utilized as the direct input for differential expression analysis within the DESeq2 package in R v4.5.2. Within the default DESeq2 pipeline, the median of ratios method was applied for count normalization, and empirical Bayes shrinkage was utilized for dispersion estimation. To visually display general gene expression abundance, fragments per kilobase of transcript per million mapped reads were calculated independently. Differentially expressed genes were identified based on the criteria of an adjusted *p* value < 0.05 and |log2FC| ≥ 1 [14] (information are detailed in Table S5). Functional annotation was carried out using DIAMOND software v2.1.8, and the significance threshold for both Gene Ontology (GO) and Kyoto Encyclopedia of Genes and Genomes (KEGG) enrichment analyses was set at *p* < 0.05 [15].

Pearson correlation coefficients were calculated between phenylpropanoid pathway DEGs and anthocyanin-related DAMs. To capture intra-group biological variance, correlations were computed across all six individual biological replicates (n = 3) rather than group means. Raw *p*-values were adjusted for multiple comparisons using the Benjamini–Hochberg FDR method. Significant gene–metabolite interactions were defined by a stringent threshold of Pearson’s *r* > 0.9 and an FDR-adjusted *p* < 0.01.

### 2.6. Bioinformatics Analysis

Multiple sequence alignments and homology searches were completed using DNAMAN v10 and online BLAST tools (https://blast.ncbi.nlm.nih.gov, accessed on 4 July 2026), including BLASTN and BLASTP, respectively [16]. Conserved domains were predicted through the NCBI Conserved Domains database [17]. The physicochemical properties and hydrophilicity of the proteins were evaluated using the ProtParam and ProtScale online tools on the ExPASy platform (https://www.expasy.org/, accessed on 4 July 2026) [18]. Secondary and tertiary structures of the proteins were modeled using SOPMA software v6.6.0 and the SWISS MODEL platform (https://swissmodel.expasy.org/, accessed on 4 July 2026), respectively [19,20]. A phylogenetic tree was constructed utilizing MEGA 11 software v11.0.13 based on the ClustalW multiple sequence alignment results [21]. Gene structure diagrams were generated by GSDS v2.0 [22]. Promoter cis acting elements were analyzed via the PlantCARE online database (http://bioinformatics.psb.ugent.be/webtools/plantcare, accessed on 4 July 2026), and subcellular localization was preliminarily predicted utilizing the WoLF PSORT tool (https://wolfpsort.hgc.jp, accessed on 4 July 2026) [23,24].

### 2.7. Tissue-Specific and Spatiotemporal Expression Analysis of Genes

Total RNA was extracted and cDNA was synthesized from various sweetpotato tissues, including young leaves, mature leaves, leaf petioles, stems, fibrous roots, pencil roots, and storage roots, as well as from storage roots at 40, 70, 100, and 130 days after field planting. Using *IbARF* as an internal reference gene, quantitative real time PCR was executed utilizing the SYBR Green quantitative fluorescence reaction system (TOYOBO Life Science, Osaka, Japan). Reaction parameters followed the kit instructions. Relative expression levels were quantified using the 2^−ΔΔCT^ method across three independent biological and technical replicates.

### 2.8. Subcellular Localization

To elucidate the spatial distribution of the IbCCoAOMT7 protein within the cell, which is crucial for understanding its functional role in the phenylpropanoid metabolic network, subcellular localization analysis was performed. The pCAMBIA1300-35S-IbCCoAOMT7-eGFP fusion vector was constructed using the homologous recombination method. To accurately validate the subcellular compartments and eliminate the possibility of free GFP diffusion, the nucleus was explicitly stained utilizing DAPI while the actin cytoskeleton was visualized utilizing the fABD-RFP marker. Subsequently, this vector was transformed into *Agrobacterium tumefaciens* strain GV3101 and injected into *Nicotiana benthamiana* leaves for transient expression, which was then observed utilizing a laser scanning confocal microscope [25].

### 2.9. Generation of Transgenic Sweetpotato

To functionally validate the in vivo biological role of *IbCCoAOMT7* in regulating the metabolic flux between lignin and anthocyanin biosynthesis, transgenic sweetpotato lines were generated. A recombinant overexpression vector pCAMBIA1301s-IbCCoAOMT7 was constructed via homologous recombination. This vector was transformed into Agrobacterium tumefaciens strain EHA105. Genetic transformation was conducted using Agrobacterium-mediated transformation with callus from Xu Zishu 8 (XZ8) serving as the receptor, which has been widely adopted as a reliable model system for sweetpotato functional genomics, overcoming the severe bottleneck of genetic transformation in this species. Transgenic lines were obtained following antibiotic selection and molecular identification according to PCR and RT-qPCR (Figure S2).

### 2.10. Determination of Total Lignin Content

Total lignin content in the storage roots was determined utilizing the acetyl bromide method. The brief procedures are as follows [26]. A 1 mg aliquot of the pretreated sample was mixed with 100 µL of an acetyl bromide and glacial acetic acid solution (25%), and reacted at 50 °C for 2 h. After cooling, 400 μL of a 2 M sodium hydroxide solution and 70 μL of a 0.5 M hydroxylamine hydrochloride solution were sequentially added to the reaction mixture, and the volume was adjusted to 2 mL with glacial acetic acid. A 200 μL aliquot of the reaction solution was transferred to a UV microplate, and the absorbance at 280 nm was measured utilizing a microplate reader. Based on the Beer Lambert law, the lignin content was calculated using the specific extinction coefficient of the lignin acetylation product at 280 nm (15.69 L·g^−1^·cm^−1^).

### 2.11. Data Processing and Statistical Analysis

All statistical analyses were performed using GraphPad Prism 10 (GraphPad Software, San Diego, CA, USA). For comparisons between two independent groups, a two-tailed Student’s *t*-test was utilized. For evaluating differences among three or more groups, a one-way analysis of variance (ANOVA) was conducted, followed by Tukey’s honestly significant difference (HSD) post hoc test for multiple comparisons. Differences were considered statistically significant at *p* < 0.05 or *p* < 0.01.

## 3. Results

### 3.1. Quantification of Total Anthocyanin Content in XZ13 and XZ13M Storage Roots

**Figure 1:**
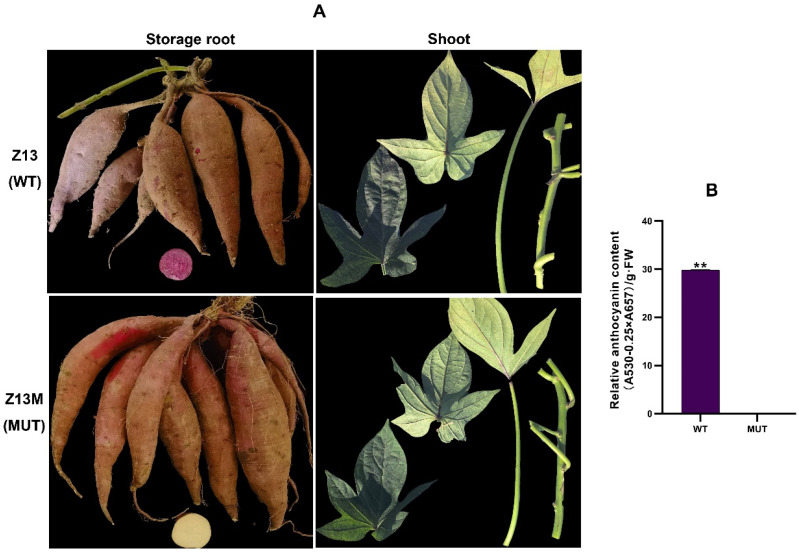
Phenotypic characterization and anthocyanin quantification of XZ13 and XZ13M. (**A**) Phenotypic comparison of the storage roots and aerial organs between XZ13 and XZ13M at the 120 days mature stage; (**B**) determination of total anthocyanin content in the storage roots of XZ13 and XZ13M. Data are presented as the mean ± standard deviation (SD, n = 3) from three biological replicates. Bars labeled with ** indicate significant differences (*p* < 0.01) according to Student’s *t*-test.

Extensive multi-year and multi-location field evaluations confirmed the stable inheritance of phenotypic traits in XZ13 and its mutant XZ13M. Phenotypic characterization of mature storage roots harvested 120 days post-planting revealed that XZ13 roots exhibited deep purple skin and flesh. Conversely, the mutant displayed a distinct phenotype characterized by red skin and yellowish flesh. Notably, no discernible morphological differences were observed in the aerial organs (leaves and stems) between the two genotypes (Figure 1A). To determine whether this variation in flesh color corresponded to altered pigmentation levels, we quantified the total anthocyanin content in the storage roots. This analysis revealed a significant reduction in anthocyanin accumulation in the mutant compared with the XZ13.

### 3.2. Metabolomic Profiling of Anthocyanin Composition and Abundance in XZ13 and XZ13M Storage Roots

**Figure 2:**
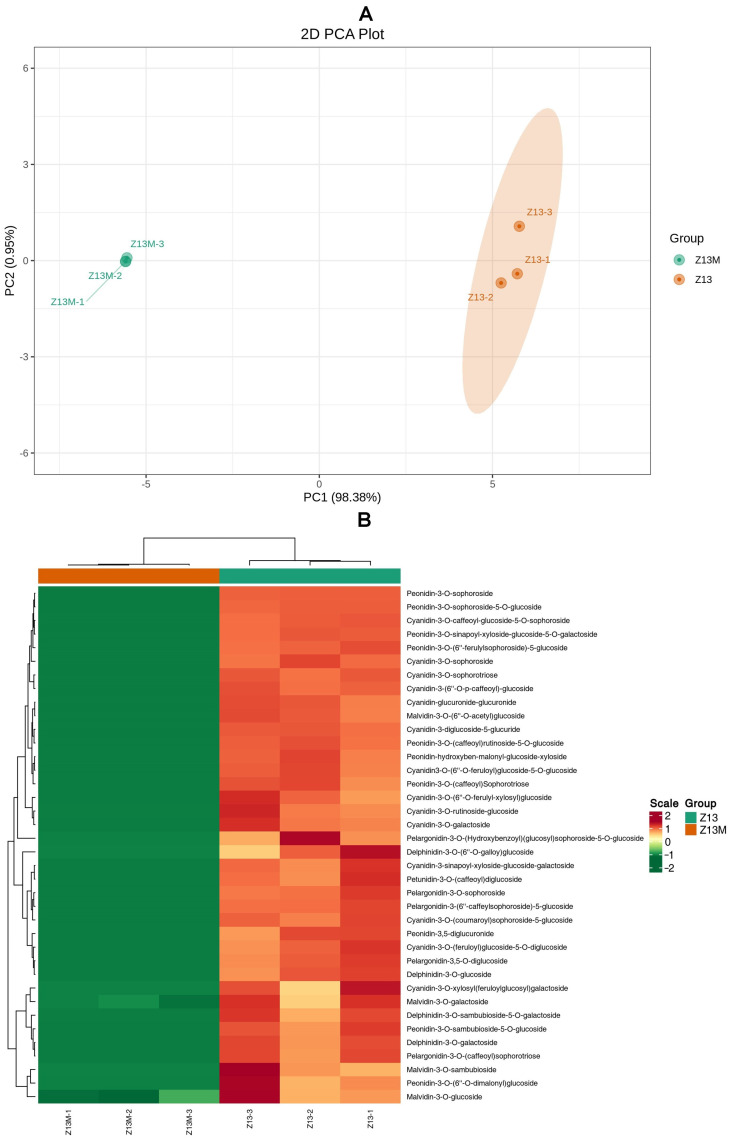

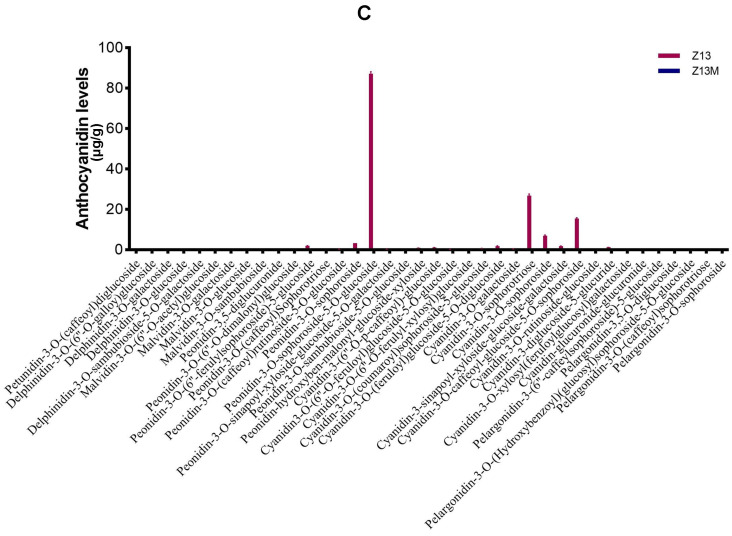
Targeted anthocyanin metabolomic analyses of XZ13 and XZ13M storage roots. (**A**) Principal component analysis based on the abundances of the 38 identified anthocyanin metabolites (the confidence ellipse for the XZ13M group is visually imperceptible because the extremely high consistency among its biological replicates results in a calculated ellipse area smaller than the data point markers); (**B**) ierarchical clustering heatmap of anthocyanin components in the storage roots of XZ13 and XZ13M. Rows represent individual metabolites, and columns represent biological replicates. The color intensity reflects the relative abundance of the metabolites; (**C**) comparison of the relative contents of high abundance differentially accumulated anthocyanin components between the storage roots of XZ13 and XZ13M. Absolute quantitative levels of core anthocyanin derivatives in storage roots calculated via standard curves and expressed as μg/g fresh weight. Data are presented as the mean ± standard deviation (SD, n = 3).

To elucidate the metabolic shifts underlying the phenotypic variation between XZ13 and XZ13M, targeted metabolomic profiling of anthocyanins was performed on the storage roots of both genotypes. Principal component analysis (PCA) revealed a clear separation between XZ13 and XZ13M’s metabolic profiles (Figure 2A). Hierarchical clustering further corroborated this divergence (Figure 2B), indicating a profound remodeling of the anthocyanin metabolic network in mutant storage roots.

In total, 38 anthocyanin metabolites were identified and quantified in XZ13 storage roots. Based on their core aglycone structures, these compounds were categorized into six major classes. Cyanidin (14 derivatives) and peonidin (10 derivatives) were predominant, followed by pelargonidin (5), delphinidin (4), malvidin (4), and petunidin (1). Conversely, anthocyanin biosynthesis in the mutant was globally impaired, with only trace amounts of three derivatives (one peonidin and two malvidin) detected. These specific residual compounds, identified as Peonidin-3-O-sophoroside-5-O-glucoside, Malvidin-3-O-glucoside and Malvidin-3-O-galactoside, were present at levels barely exceeding the quantification limits, underscoring the severe metabolic bottleneck in the mutant.

Differential analysis demonstrated that all 38 anthocyanins exhibited significantly altered accumulation between the two genotypes (Table S1), characterized by a near-complete depletion in the mutant. Specifically, XZ13 roots primarily accumulated highly glycosylated and acylated derivatives. Among these, peonidin-3-O-sophoroside-5-O-glucoside, cyanidin-3-O-sophoroside, peonidin-3-O-sophoroside, cyanidin-3-O-sophorotriose, and cyanidin-3-O-caffeoyl-glucoside-5-O-sophoroside exhibited the highest relative abundances (Figure 2C).

### 3.3. Transcriptomic Profiling and Identification of Differentially Expressed Genes Between XZ13 and XZ13M

Following stringent quality control, six independent cDNA libraries yielded between 50,956,956 and 84,376,162 high-quality clean reads. These reads were subsequently mapped to the sweetpotato reference genome, achieving alignment rates of 98.00% to 98.60%. Furthermore, Q20 and Q30 scores for each library consistently exceeded 97.89% and 93.49%, respectively, whilst GC content remained at approximately 45% (Table S2). These metrics confirmed the high reliability of the RNA-seq dataset.

**Figure 3:**
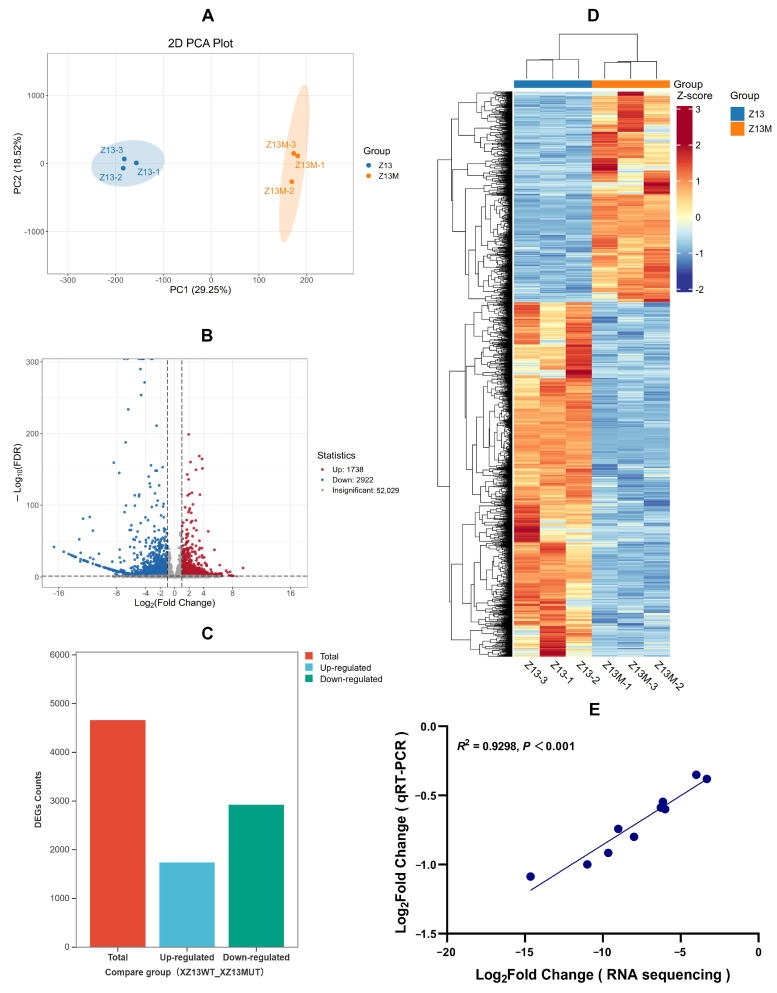
Transcriptomic analyses of the storage roots of XZ13 and XZ13M. (**A**) Principal component analysis results based on global gene expression profiles; (**B**,**C**) volcano plot and bar graph displaying the statistical counts of differentially expressed genes; (**D**) hierarchical clustering heatmap of the 4660 DEGs. Rows represent different differentially expressed genes, and columns represent biological replicate samples. The color gradient from blue to red indicates the relative gene expression levels; (**E**) correlation analysis of expression levels between RNA sequencing and RT-qPCR for 10 core DEGs associated with anthocyanin biosynthesis.

PCA of global gene expression profiles revealed clear separation between XZ13 and XZ13M (Figure 3A), indicating substantial transcriptional reprogramming in the mutant storage roots. We further identified 4660 differentially expressed genes (DEGs) between the two genotypes, comprising 1738 up- and 2922 downregulated genes (Figure 3B,C). Notably, the predominance of downregulated genes aligned with the observed large-scale repression of the anthocyanin biosynthetic pathway in the mutant. Hierarchical clustering further confirmed the contrasting expression patterns of these DEGs between genotypes (Figure 3D), reflecting the extensive and specific transcriptional responses associated with the color mutation.

To validate the technical accuracy of the RNA-seq quantification, 10 DEGs integral to anthocyanin biosynthesis were selected for independent RT-qPCR analysis. The relative expression levels obtained via RT-qPCR exhibited a significant positive correlation with the RNA-seq data (Figure 3E). This high degree of consistency confirmed the robustness of the transcriptomic dataset for further dissecting the anthocyanin metabolic regulatory network.

### 3.4. Functional Annotation and Metabolic Pathway Enrichment Analysis of DEGs

**Figure 4:**
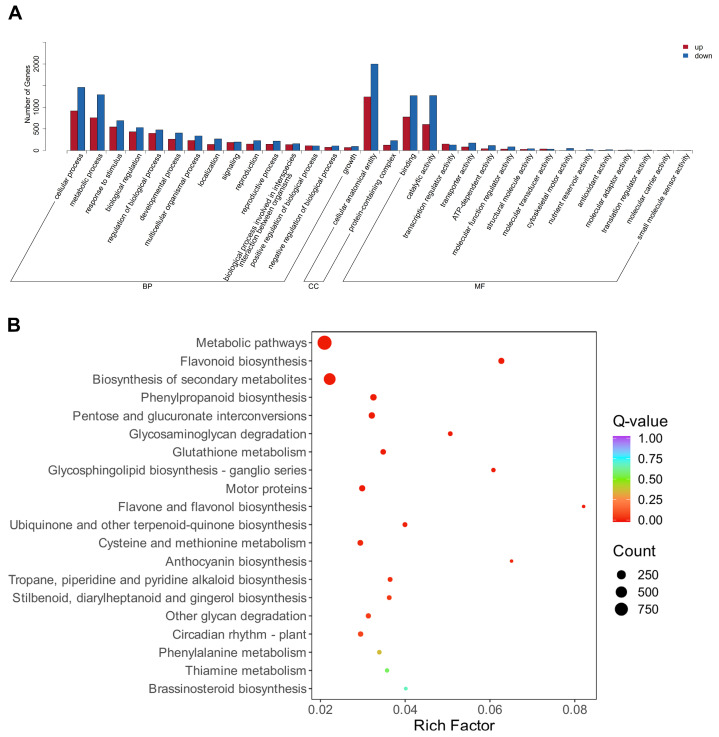
Functional annotation and metabolic pathway enrichment analyses of differentially expressed genes in the storage roots of XZ13 and XZ13M. (**A**) Gene Ontology functional classification statistics of the DEGs. The horizontal axis represents the specific Gene Ontology functional subcategories, and the vertical axis indicates the number of DEGs annotated to each subcategory; (**B**) bubble plot of KEGG metabolic pathway enrichment analysis for the differentially expressed genes. The vertical axis represents the names of significantly enriched metabolic pathways, and the horizontal axis represents the enrichment factor. The size of the bubbles corresponds to the number of DEGs enriched in the pathway, and the color gradient indicates the statistical significance of the enrichment.

The DEGs were mapped to 32 functional subcategories according to GO annotation, comprising 15 biological processes, two cellular components, and 15 molecular functions. Terms including ‘cellular process’, ‘metabolic process’, ‘response to stimulus’, ‘cellular anatomical entity’, ‘binding’, and ‘catalytic activity’ were the most highly represented (Figure 4A). This profile indicated that mutant storage roots underwent extensive remodeling of basal physiological and metabolic processes during development.

Twenty-four pathways were significantly enriched according to KEGG pathway enrichment analysis (Figure 4B), encompassing essential regulatory circuits such as plant–pathogen interaction, plant hormone signal transduction, and starch and sucrose metabolism. Notably, a cascade of pathways directly governing anthocyanin biosynthesis exhibited highly significant enrichment in the mutant. These included the biosynthesis of secondary metabolites (ko01110), phenylpropanoid biosynthesis (ko00940), flavonoid biosynthesis (ko00941), flavone and flavonol biosynthesis (ko00944), and anthocyanin biosynthesis (ko00942). This transcriptional enrichment signature closely corroborated our targeted metabolomic data, confirming the pronounced depletion of anthocyanin metabolites.

### 3.5. Expression Profiling of the Phenylpropanoid Pathway and Identification of IbCCoAOMT

**Figure 5:**
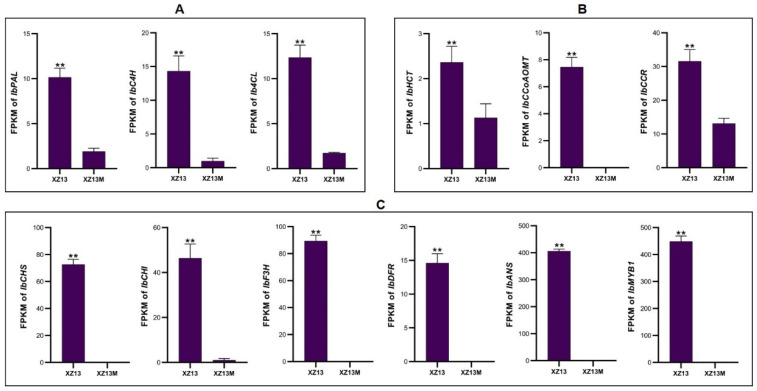
Transcriptional expression patterns of key genes in the phenylpropanoid metabolic network in the storage roots of XZ13 and XZ13M. (**A**) Relative expression levels of global upstream structural genes in the phenylpropanoid pathway; (**B**) relative expression levels of specific key genes in the lignin biosynthetic branch; (**C**) relative expression levels of structural genes in the anthocyanin biosynthetic branch and the core transcription factor *IbMYB1*. The vertical axis represents mean FPKM values calculated from RNA sequencing data. Data are presented as the mean ± standard deviation (SD, n = 3) from three biological replicates. Bars labeled with ** indicate significant differences (*p* < 0.01) according to Student’s *t*-test.

To systematically investigate the molecular mechanisms underlying the blockade of anthocyanin biosynthesis in the purple-fleshed sweetpotato mutant, we analyzed the phenylpropanoid biosynthesis pathway (ko00940), the central hub providing precursors for both the flavonoid, anthocyanin (ko00941, ko00942), and lignin branches. Comparative transcriptomic analyses revealed that the expression of early structural genes within this pathway was systemically repressed in XZ13M. Relative to the XZ13, the transcript abundances of *IbPAL*, *IbC4H*, and *Ib4CL*, which facilitate the entry of precursors into the pathway, were significantly reduced (Figure 5A). This suggested that the total carbon flux directed into the phenylpropanoid metabolic network was severely limited in the mutant.

Following the metabolic flux downstream, we observed that the anthocyanin biosynthetic branch in the mutant was virtually inactive. The expression of key structural genes, including *IbCHS*, *IbCHI*, *IbF3H*, *IbDFR*, and *IbANS*, exhibited a precipitous decline in XZ13M, with several transcripts being nearly undetectable (Figure 5C). Furthermore, the expression of *IbMYB1*, a master transcription factor regulating anthocyanin biosynthesis in sweetpotato, was also significantly suppressed.

Notably, the lignin biosynthetic branch exhibited a coordinated transcriptional attenuation. The expression of genes associated with lignin monomer synthesis, such as *IbHCT* and *IbCCR*, was significantly downregulated in the mutant (Figure 5B). Amongst these differentially expressed genes, the profile of *IbCCoAOMT* was particularly noteworthy. Positioned at a critical bifurcation point for lignin monomer synthesis, *IbCCoAOMT* retained robust expression in XZ13 to maintain metabolic homeostasis, whilst its transcript levels were nearly abolished in the XZ13M (Figure 5B).

To integrate the transcriptomic and metabolomic datasets and pinpoint the core regulators, we constructed a gene–metabolite correlation matrix using Pearson correlation analysis between the phenylpropanoid pathway DEGs and the anthocyanin-related differentially accumulated metabolites (DAMs). To filter the candidates, a stringent statistical threshold (Pearson coefficient *r* > 0.9, *p* < 0.01) was applied. This mathematical integration generated a subset of highly connected candidate genes, among which *IbCCoAOMT* exhibited one of the strongest positive correlations (*r* > 0.99) with the abundance of core anthocyanin constituents that were substantially depleted in the mutant, specifically including peonidin-3-*O*-sophoroside-5-*O*-glucoside, cyanidin-3-*O*-sophoroside, cyanidin-3-*O*-sophorotriose, and cyanidin-3-*O*-caffeoyl-glucoside-5-*O*-sophoroside (Table S3). Biologically, the profound downregulation of *IbCCoAOMT7*, alongside anthocyanin loss in the XZ13M, likely represents a correlated consequence of global repression across the upstream phenylpropanoid pathway, rather than the initial cause of pigment depletion. To avoid logical ambiguity, we must explicitly distinguish this background correlation from the intrinsic regulatory capacity of the gene. Unlike downstream structural genes, *IbCCoAOMT7* occupies a critical topological bifurcation node within the lignin branch. Therefore, to rigorously evaluate its specific functional effect when overexpressed, independent of system-wide repression, we selected *IbCCoAOMT7* as the primary candidate for subsequent transgenic functional validation regarding metabolic flux allocation.

### 3.6. Sequence Characteristics and Evolutionary Analysis of the IbCCoAOMT

Sequence similarity analysis revealed that the IbCCoAOMT amino acid sequence shares the highest homology with AtCCoAOMT7 in *Arabidopsis thaliana*, and the gene was designated as IbCCoAOMT7. Physicochemical analysis of the IbCCoAOMT7 amino acid sequence revealed a theoretical molecular weight of 25.87 kDa and an isoelectric point (pI) of 4.98, indicating a weakly acidic nature. The protein exhibited an instability index of 39.74 and a grand average of hydropathicity (GRAVY) score of −0.071, suggesting it is a stable, soluble, and hydrophilic protein. Subcellular localization predictions indicated that IbCCoAOMT7 is primarily localized to the cytoplasm. Secondary structure analysis demonstrated that the conformational backbone predominantly comprises alpha helices (40.69%) and random coils (40.69%), with extended strands accounting for only 18.61%.

**Figure 6:**
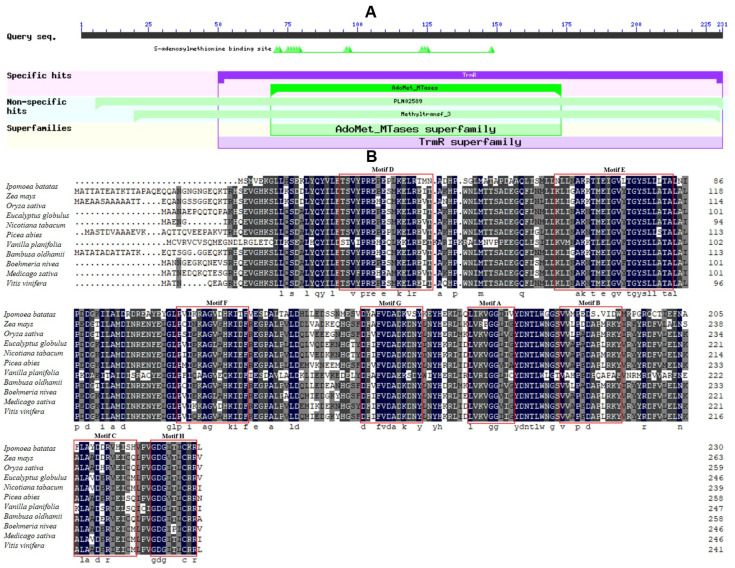
Sequence characteristics and structural analysis of IbCCoAOMT7. (**A**) Schematic diagram of the predicted conserved domains of the IbCCoAOMT7 protein; (**B**) multiple amino acid sequence alignment of IbCCoAOMT7 with homologous CCoAOMT proteins from other representative plant species. Different background color intensities indicate the levels of identity and similarity among the amino acid residues. Identical and similar amino acid residues are highlighted with dark and grey shading, respectively, whereas unshaded regions indicate non-conserved residues. The conserved motifs are delineated by red boxes.

Phylogenetic analysis of plant CCoAOMT proteins (Figure S1) demonstrated that IbCCoAOMT7 shares a highly conserved evolutionary trajectory. It clustered closely with *Ipomoea nil*, indicating the closest genetic relationship, followed by *Capsicum baccatum* and *Capsicum chinense*. Conserved domain analysis subsequently confirmed that IbCCoAOMT7 harbors a typical AdoMet_MTases catalytic domain (Figure 6A), firmly classifying it within the S-adenosylmethionine-dependent methyltransferase superfamily.

To elucidate the structural basis of its substrate-binding and catalytic activity, a multiple sequence alignment (MSA) was performed against 10 representative plant CCoAOMT homologs. This analysis revealed that despite some divergence at the N-terminus, the overall backbone and core catalytic regions remained highly conserved (Figure 6B). Crucially, IbCCoAOMT7 not only retains the core substrate-binding motifs (Motifs A, B, and C) characteristic of plant S-adenosylmethionine-dependent methyltransferases, but also harbors the five signature motifs (Motifs D, E, F, G, and H) essential for specific CCoAOMT function. The presence of these conserved domains and key motifs strongly supports its putative role as a highly specific catalyst for lignin monomer methylation.

### 3.7. Tissue Expression Pattern and Subcellular Localization Analysis of IbCCoAOMT7

**Figure 7:**
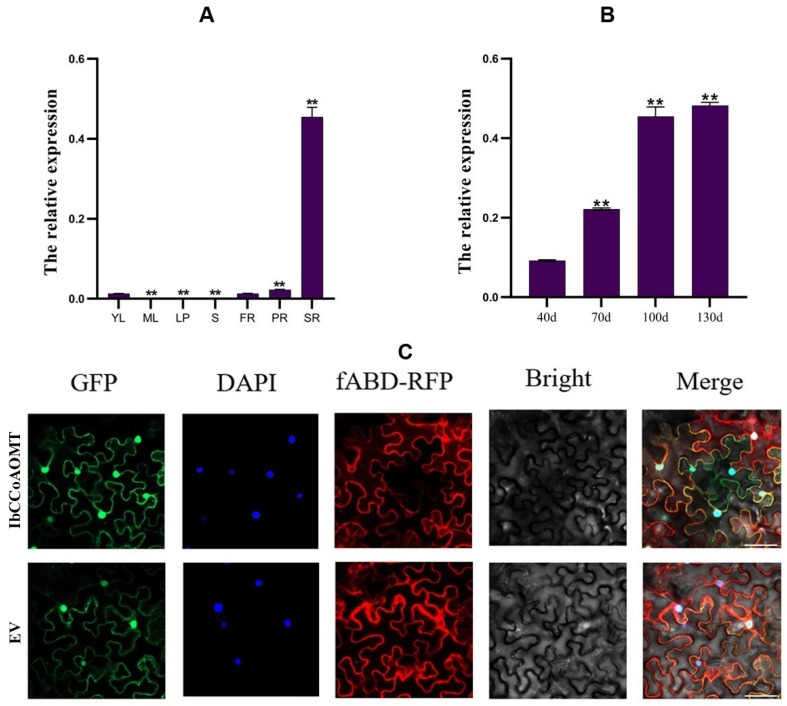
Spatiotemporal expression patterns and subcellular localization analysis of IbCCoAOMT7. (**A**) Relative expression levels of the *IbCCoAOMT7* in various sweetpotato tissues and organs, specifically young leaf (YL), mature leaf (ML), leaf petiole (LP), stem (S), fibrous root (FR), pencil root (PR), and storage root (SR); (**B**) relative expression levels of the *IbCCoAOMT7* at various stages of storage root development, with 40, 70, 100, and 130 days representing different growth phases after planting; (**C**) transient subcellular localization of the IbCCoAOMT7 protein in leaf epidermal cells of *Nicotiana benthamiana*. Scale bar = 25 μm. Data are presented as the mean ± standard deviation (SD, n = 3) from three biological replicates. Bars labeled with ** indicate significant differences (*p* < 0.01) according to ANOVA followed by Dunnett’s multiple comparisons test.

To elucidate the functional role of *IbCCoAOMT7* during sweetpotato development, RT-qPCR was employed to analyze its spatiotemporal expression profile across various tissues and developmental stages. Whilst ubiquitously expressed throughout the plant, *IbCCoAOMT7* exhibited distinct tissue-specific preferentiality (Figure 7A). Transcript abundance was highest in storage roots, followed by pencil roots, whereas moderate expression was maintained in fibrous roots and young leaves. Conversely, expression levels in aerial vegetative organs (mature leaves, petioles, and stems) remained exceedingly low. Furthermore, developmental profiling of roots at 40, 70, 100, and 130 days after planting (DAP) revealed that *IbCCoAOMT7* expression increased rapidly during the early-to-mid stages of expansion (40 to 70 DAP), plateauing at a high level during late development (100 to 130 DAP) (Figure 7B). This spatiotemporal pattern indicates that phenylpropanoid metabolic flux, centered on *IbCCoAOMT7*, is heavily activated during storage root initiation and rapid expansion. This robust transcriptional activity not only facilitates lignin biosynthesis to reinforce vascular tissue development but also regulates metabolic flux allocation, providing essential precursors for the subsequent accumulation of secondary metabolites such as anthocyanins.

Furthermore, to ascertain the subcellular localization of the IbCCoAOMT7 protein, confocal microscopy was conducted. In contrast to the diffuse signal produced by the empty vector control, the fluorescence signal from the IbCCoAOMT7-GFP fusion was distinctly localized to the cytoplasm and the nucleus (Figure 7C).

### 3.8. Overexpression of IbCCoAOMT7 Significantly Inhibited Anthocyanin Accumulation in Sweetpotato Storage Roots

**Figure 8:**
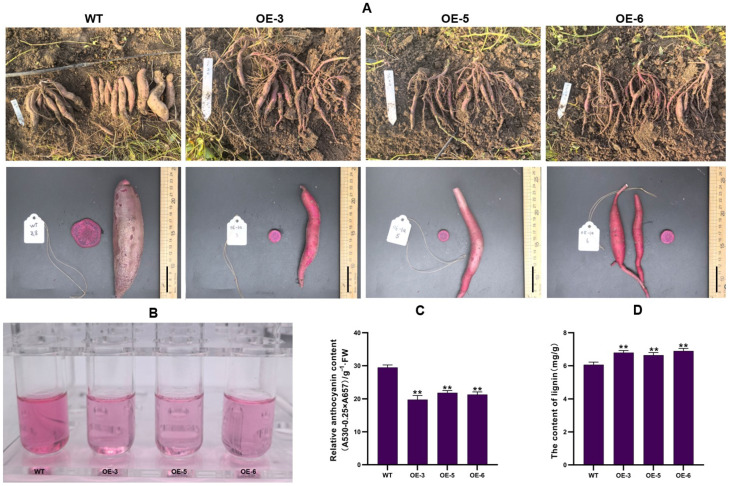
Effects of *IbCCoAOMT7* overexpression on the phenotype and metabolic flux allocation in sweetpotato storage roots. (**A**,**B**) Phenotypic comparison of the storage roots between WT and OE lines at 120 days after field planting. Bar = 4 cm; (**C**) comparison of total anthocyanin content in the storage roots of the wild type and overexpression lines; (**D**) comparison of total lignin content in the storage roots of the WT and OE line. Data are presented as the mean ± standard deviation (SD, n = 3) from three biological replicates. Bars labeled with ** indicate significant differences (*p* < 0.01) according to ANOVA followed by Dunnett’s multiple comparisons test.

Because the natural mutant primarily reflects correlative network changes, we generated *IbCCoAOMT7*-overexpressing sweetpotato lines via Agrobacterium-mediated transformation. to establish the causal regulatory role of this gene in anthocyanin accumulation. Following molecular verification, three independent transgenic lines (OE3, OE5, and OE6) were obtained. These lines, alongside the WT (XZ13XZ8) control, were cultivated in the field for 120 days prior to the harvesting and phenotypic characterization of storage roots. In contrast to the deep purple color of WT roots, all overexpression lines exhibited a pronounced loss of pigmentation (Figure 8A,B). Quantitative analysis confirmed that total anthocyanin accumulation in the OE lines was significantly reduced compared with the WT (Figure 8C), whilst lignin content was concurrently significantly elevated (Figure 8D). While these divergent phenotypic outcomes strongly suggest a redirection of carbon flux toward the lignin pathway at the expense of anthocyanin biosynthesis, we acknowledge that measuring total content alone cannot definitively prove absolute metabolic reallocation.

**Figure 9:**
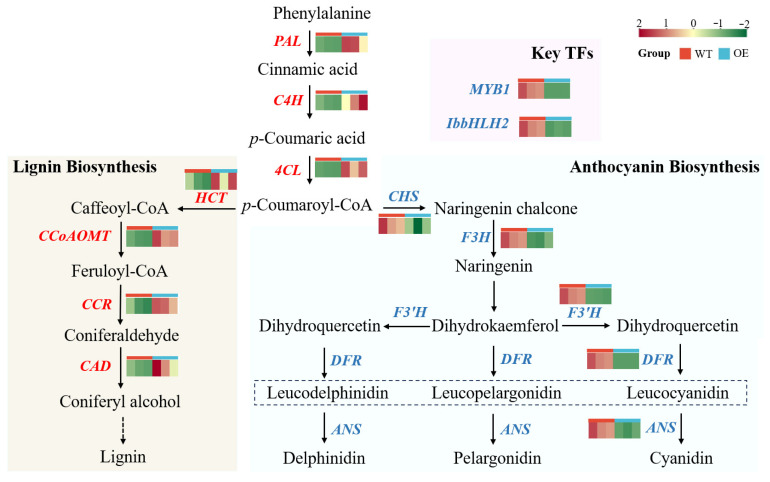
Relative expression levels of key enzyme genes in the phenylpropanoid metabolic pathway in sweetpotato storage roots overexpressing *IbCCoAOMT7*. The red color represents the WT, and the blue color represents the OE line. The color intensity reflects the relative gene expression levels, where the color scale from −2 to 2 represents row standardized log_2_ transformed values.

Among the generated independent transgenic lines, the OE-3 line was characterized by the most significant reduction in anthocyanin accumulation and a pronounced increase in lignin accumulation, which was selected as the typical representative line to explore the underlying molecular mechanisms. The results demonstrated that *IbCCoAOMT7* overexpression triggered a systemic transcriptional repression of the anthocyanin-specific branch, with structural genes (*IbCHS*, *IbF3H*, *IbF3’H*, *IbDFR*, and *IbANS*) exhibiting comprehensive and significant downregulation (Figure 9). Crucially, the transcript abundances of *IbMYB1* and *IbbHLH2*, the master transcriptional activators governing this pathway, were similarly substantially decreased. Conversely, early structural genes of the general phenylpropanoid pathway (*IbPAL*, *IbC4H*, and *Ib4CL*) and key genes of the lignin-specific branch (*IbHCT*, *IbCCR*, and *IbCAD*) were significantly upregulated in the OE line.

## 4. Discussion

### 4.1. Transcriptional Reprogramming of the Phenylpropanoid Metabolic Network Resulted in the Natural Variation in Sweetpotato Storage Root Coloration

Anthocyanins are key metabolites dictating the nutritional quality and commercial value of purple-fleshed sweetpotato. Our targeted metabolomic data confirmed that the loss of purple-red coloration in XZ13M storage roots resulted directly from a comprehensive depletion of glycosylated and acylated anthocyanins, primarily those centered on cyanidin and peonidin. Natural variation in plant coloration typically arises via two mechanisms: the functional mutation of a single structural gene, or the comprehensive shutdown of a pathway driven by a transcriptional regulatory network [27]. Transcriptomic evidence from the present study clearly indicated that anthocyanin depletion in the mutant was accompanied by the systemic transcriptional repression of the core phenylpropanoid pathway, including PAL, C4H, and 4CL. This demonstrates that the drastic variation in storage root coloration in the XZ13 mutant results from a global reprogramming of the secondary metabolic network.

From a metabolic perspective, this global suppression represents a biological imperative. The phenylpropanoid pathway is highly energy-demanding. The isolated inactivation of a downstream terminal gene would not only waste carbon resources but also risk the abnormal accumulation of intermediates, such as cinnamic acid, potentially triggering cytotoxicity [28]. Consequently, plants tend to restrict carbon flux at the source by deactivating master regulatory switches—a phenomenon widely documented in natural variants of species such as grape and apple [29,30]. Our findings perfectly align with this established paradigm.

Against this backdrop of global transcriptional remodeling, we pinpointed *IbCCoAOMT7* as a critical diversion-node gene tightly correlated with anthocyanin accumulation. In the WT, this gene was highly expressed to support vascular tissue development. Conversely, in the mutant, because upstream metabolic flux was restricted at the source, the profound suppression of *IbCCoAOMT7* likely represented a downstream consequence of this overarching global repression. This observation not only confirms that substrate limitation is a prerequisite for pigment loss but also offers a novel perspective. Given its role as a key enzyme at a metabolic branch point, *IbCCoAOMT7* transcript abundance is intrinsically linked to upstream flux supply. This raises a compelling hypothesis: under normal physiological conditions with replete substrates, could the robust activation of this enzyme act as a rate-limiting ‘diversion valve’, actively sequestering carbon flux away from the anthocyanin pathway to inhibit pigment accumulation? Driven by this scientific premise, we subsequently undertook the functional characterization of *IbCCoAOMT7*.

### 4.2. IbCCoAOMT7 Negatively Regulates Anthocyanin Accumulation by Competing for Common Substrates

Within the branched phenylpropanoid pathway, carbon flux allocation is subject to precise and dynamic homeostatic regulation. Here, the early bifurcation node between lignin and flavonoid/anthocyanin biosynthesis is of paramount importance [31]. These two branches heavily rely on and compete for a shared pool of precursors, notably *p*-coumaroyl-CoA and caffeoyl-CoA. In the present study, overexpressing *IbCCoAOMT7*, a key rate-limiting enzyme in lignin biosynthesis, led to a surge in lignin content alongside a dramatic reduction in anthocyanins in sweetpotato storage roots. This reciprocal relationship at the substrate level aligns with metabolic flux redirection mechanisms reported across multiple species. However, further direct quantification of relevant intermediate metabolites remains necessary to definitively prove this specific biochemical redistribution. Extensive reverse genetics studies confirm that blocking the lignin branch effectively redirects carbon flux into the anthocyanin pathway. For instance, silencing *HCT* or *CCR* in Arabidopsis induces abnormal purplish-red pigmentation in stems [5,8]. Similarly, in strawberry, downregulating the peroxidase *FaPRX27* significantly deepens fruit coloration [31]. Compelling functional evidence has been observed in *Petunia*, where the targeted suppression of a *CCoAOMT* homolog prevented the conversion of caffeoyl-CoA to lignin monomers, thereby redirecting the metabolic flux toward anthocyanin hyperaccumulation [32]. Collectively, these findings demonstrate that the activity of rate-limiting enzymes at metabolic bifurcations plays a highly conserved and decisive role in partitioning plant secondary metabolites.

The current study highlights the role of *IbCCoAOMT7* as a rate-limiting ‘diversion valve’ within the sweetpotato secondary metabolic network. When constitutively overexpressed, the robust catalytic activity of IbCCoAOMT7 establishes a dominant metabolic sink within the cytoplasm. This disrupts the endogenous carbon allocation homeostasis of the storage root, channeling shared substrates towards feruloyl-CoA synthesis. Consequently, it outcompetes chalcone synthase for carbon flux via substrate competition [33]. This substrate-driven sink effect not only elucidates the biochemical and metabolic mechanisms underlying the loss of pigmentation in the overexpression lines but also provides a strategic framework for the molecular breeding of nutritionally enhanced sweetpotato. When developing anthocyanin-rich varieties, finely balancing the carbon flux competition between lignin-supported vascular development and anthocyanin accumulation is essential, while *IbCCoAOMT7* emerges as a pivotal genetic target.

### 4.3. Hypothesized Mechanisms of Negative Feedback and Transcriptional Repression in Anthocyanin Biosynthesis

The negative regulatory effect of *IbCCoAOMT7* overexpression on anthocyanin accumulation extends beyond mere substrate depletion. Molecular evidence from the present study demonstrated that in the overexpression lines, downstream structural genes of the anthocyanin pathway were significantly downregulated, and the expression of core regulatory factors (*IbMYB1* and *IbbHLH2*) driving sweetpotato anthocyanin biosynthesis was severely repressed. While our current dataset lacks direct biochemical proof, we propose two hypothetical mechanisms to explain this transcriptional suppression. Firstly, we hypothesize that this transcriptional suppression may stem from two distinct mechanisms. Firstly, the hyperactivation of the lignin branch likely results in the abnormal accumulation of specific phenylpropanoid intermediates, such as cinnamic acid derivatives. Acting as retrograde signaling molecules, these metabolites might exert negative feedback inhibition on the anthocyanin-specific MBW protein complex. As previously demonstrated in *Arabidopsis*, the excessive accumulation of cinnamic acid can specifically repress the expression of core *MYB* factors, including *PAP1*, thereby attenuating their transcriptional activation of the anthocyanin pathway [34]. As a hypothesized theoretical model, the dual subcellular localization of the IbCCoAOMT7 protein observed in our study implies that this methyltransferase might not only execute catalytic functions within the cytoplasm, but could also potentially acts as a ‘moonlighting’ protein. We conservatively hypothesize that it might mediate transcriptional repression within the nucleus. This proposed non-canonical hypothesis aligns with a recently elucidated mechanism in *Arabidopsis*, wherein the AtCCoAOMT1 protein shuttles into the nucleus to finely modulate the abundance and activity of specific *bHLH* transcription factors via physical interaction [35]. Ultimately, we emphasize that these dual mechanisms remain strictly theoretical within the context of our current dataset. Future empirical investigations are absolutely required to rigorously test these hypotheses. These planned future studies will involve targeted intermediate quantification, yeast two-hybrid (Y2H), bimolecular fluorescence complementation (BiFC) with IbMYB1 or IbbHLH2, and dual-luciferase reporter assays. Nevertheless, our current findings provide a preliminary theoretical framework for elucidating the intricate crosstalk between the divergent lignin and anthocyanin branches.

## 5. Conclusions

By integrating metabolomic, transcriptomic, and reverse genetics approaches, this study provides a systemic elucidation of the molecular mechanisms underlying differential anthocyanin accumulation in purple-fleshed sweetpotato. We identified *IbCCoAOMT7* as a potential hub gene associated with metabolic flux partitioning within the phenylpropanoid network. Our findings indicate that anthocyanin deficiency in storage roots is strongly correlated with a widespread transcriptional repression of the phenylpropanoid and flavonoid pathways. *IbCCoAOMT7* is suggested to function as a promising candidate affecting the lignin–anthocyanin balance, rather than a definitively proven regulator of redirected carbon flux. Its upregulation is associated with an increased accumulation of lignin and a concomitant suppression of anthocyanin accumulation parallel to the downregulation of the regulatory factors *IbMYB1* and *IbbHLH2*. However, because our current evidence is derived from correlative multi-omics data and transgenic phenotypes, direct metabolic flux validation remains necessary to definitively establish absolute carbon flux reallocation within sweetpotato storage roots. Collectively, this research provides novel insights into the potentially coordinated regulatory mechanisms governing multi-branched secondary metabolic networks in plants, offering critical genetic targets for the precision breeding of sweetpotato varieties with enhanced nutritional and functional properties.

## Data Availability

The original contributions presented in this study are included in the article/Supplementary Materials. Further inquiries can be directed to the corresponding authors.

## Supplementary Materials

The following supporting information can be downloaded at: https://www.mdpi.com/article/10.3390/biology15141102/s1, Figure S1: Phylogenetic tree of IbCCoAOMT protein from sweetpotato and CCoAOMT proteins from other species; Figure S2: Comprehensive molecular validation of the generated IbCCoAOMT7 transgenic lines. Table S1: Determination and Quantification of Anthocyanins in Species; Table S2: Quality Control Information for Transcriptome Sequencing; Table S3: Correlation analysis between DEGs and DAMs; Table S4: Sequences of qRT-PCR Detection and PCR Amplification Primers; Table S5: Relative Expression Levels of Structural Genes in Phenylaprapanoid metabolism; Table S6: Linear regression equations and correlation coefficients of standard curves for absolute quantification of anthocyanin metabolites.

## References

[1] Yang J., Moeinzadeh M.H., Kuhl H., Helmuth J., Xiao P., Haas S., Liu G., Zheng J., Sun Z., Fan W. (2017). Haplotype-resolved sweet potato genome traces back its hexaploidization history. Nat. Plants.

[2] Tang C., Han J., Chen D., Zong S., Liu J., Kan J., Qian C., Jin C. (2023). Recent advances on the biological activities of purple sweet potato anthocyanins. Food Biosci..

[3] Shen L., Yang Y., Zhang J., Feng L., Zhou Q. (2023). Diacylated anthocyanins from purple sweet potato (*Ipomoea batatas* L.) attenuate hyperglycemia and hyperuricemia in mice induced by a high-fructose/high-fat diet. J. Zhejiang Univ. Sci. B.

[4] Xu W., Dubos C., Lepiniec L. (2015). Transcriptional control of flavonoid biosynthesis by MYB-bHLH-WDR complexes. Trends Plant Sci..

[5] Besseau S., Hoffmann L., Geoffroy P., Lapierre C., Pollet B., Legrand M. (2007). Flavonoid accumulation in Arabidopsis repressed in lignin synthesis affects auxin transport and plant growth. Plant Cell.

[6] Zhang M., Li X., Wang X., Jiang S., Zhang J., Sun M., Zhou Z., Zhang J., Li M., Lv Y. (2025). Modulation of lignin and anthocyanin homeostasis by GTP cyclohydrolase1 in maize. Plant Biotechnol. J..

[7] Wang L., Pan D., Liang M., Abubakar Y.S., Li J., Lin J., Chen S., Chen W. (2017). Regulation of Anthocyanin Biosynthesis in Purple Leaves of Zijuan Tea (*Camellia sinensis* var. *kitamura*). Int. J. Mol. Sci..

[8] Gallego-Giraldo L., Jikumaru Y., Kamiya Y., Tang Y., Dixon R.A. (2011). Selective lignin downregulation leads to constitutive defense response expression in alfalfa (*Medicago sativa* L.). New Phytol..

[9] Lan M., Kou M., Li C., Gao Z., Liu X., Zhang Y., Wang X., Tang W., Yan H., Gao T. (2026). Sweetpotato solute carrier family 35 member F1 (IbSLC35F1), a nucleotide sugar transporter transcriptionally activated by IbMYB1, modulates anthocyanin accumulation in storage roots. Int. J. Biol. Macromol..

[10] Deikman J., Hammer P.E. (1995). Induction of anthocyanin accumulation by cytokinins in *Arabidopsis thaliana*. Plant Physiol..

[11] Zhao D., Zhao L., Liu Y., Zhang A., Xiao S., Dai X., Yuan R., Zhou Z., Cao Q. (2022). Metabolomic and Transcriptomic Analyses of the Flavonoid Biosynthetic Pathway for the Accumulation of Anthocyanins and Other Flavonoids in Sweetpotato Root Skin and Leaf Vein Base. J. Agric. Food Chem..

[12] Porebski S., Bailey L.G., Baum B.R. (1997). Modification of a CTAB DNA extraction protocol for plants containing high polysaccharide and polyphenol components. Plant Mol. Biol. Report..

[13] Kim D., Langmead B., Salzberg S.L. (2015). HISAT: A fast spliced aligner with low memory requirements. Nat. Methods.

[14] Gargano G., Esposito F., Del Buono N., Ciavarella S., Vegliante M.C. (2026). Identification of differentially expressed genes in RNA-seq data via semi-rigid orthogonal sparse KL-NMTF. BMC Bioinform..

[15] Svedberg D., Winiger R.R., Berg A., Sharma H., Tellgren-Roth C., Debrunner-Vossbrinck B.A., Vossbrinck C.R., Barandun J. (2024). Functional annotation of a divergent genome using sequence and structure-based similarity. BMC Genom..

[16] Altschul S.F., Madden T.L., Schaffer A.A., Zhang J., Zhang Z., Miller W., Lipman D.J. (1997). Gapped BLAST and PSI-BLAST: A new generation of protein database search programs. Nucleic Acids Res..

[17] Lu S., Wang J., Chitsaz F., Derbyshire M.K., Geer R.C., Gonzales N.R., Gwadz M., Hurwitz D.I., Marchler G.H., Song J.S. (2020). CDD/SPARCLE: The conserved domain database in 2020. Nucleic Acids Res..

[18] Artimo P., Jonnalagedda M., Arnold K., Baratin D., Csardi G., de Castro E., Duvaud S., Flegel V., Fortier A., Gasteiger E. (2012). ExPASy: SIB bioinformatics resource portal. Nucleic Acids Res..

[19] Geourjon C., Deleage G. (1995). SOPMA: Significant improvements in protein secondary structure prediction by consensus prediction from multiple alignments. Comput. Appl. Biosci..

[20] Waterhouse A., Bertoni M., Bienert S., Studer G., Tauriello G., Gumienny R., Heer F.T., de Beer T.A.P., Rempfer C., Bordoli L. (2018). SWISS-MODEL: Homology modelling of protein structures and complexes. Nucleic Acids Res..

[21] Tamura K., Stecher G., Kumar S. (2021). MEGA11: Molecular Evolutionary Genetics Analysis Version 11. Mol. Biol. Evol..

[22] Hu B., Jin J., Guo A.Y., Zhang H., Luo J., Gao G. (2015). GSDS 2.0: An upgraded gene feature visualization server. Bioinformatics.

[23] Lescot M., Dehais P., Thijs G., Marchal K., Moreau Y., Van de Peer Y., Rouze P., Rombauts S. (2002). PlantCARE, a database of plant cis-acting regulatory elements and a portal to tools for in silico analysis of promoter sequences. Nucleic Acids Res..

[24] Horton P., Park K.J., Obayashi T., Fujita N., Harada H., Adams-Collier C.J., Nakai K. (2007). WoLF PSORT: Protein localization predictor. Nucleic Acids Res..

[25] Tang R., Zhao C., Dong J., Liu X., Chang L., Li J., Dong H., Lv Y., Luo Z., Wu M. (2025). Post-transcriptional and post-translational regulation of anthocyanin biosynthesis in sweetpotato by Ib-miR2111 and IbKFB: Implications for health promotion. J. Adv. Res..

[26] Zhao X., Zhao Y., Gou M., Liu C.J. (2023). Tissue-preferential recruitment of electron transfer chains for cytochrome P450-catalyzed phenolic biosynthesis. Sci. Adv..

[27] Albert N.W., Davies K.M., Lewis D.H., Zhang H., Montefiori M., Brendolise C., Boase M.R., Ngo H., Jameson P.E., Schwinn K.E. (2014). A conserved network of transcriptional activators and repressors regulates anthocyanin pigmentation in eudicots. Plant Cell.

[28] Vanholme B., El Houari I., Boerjan W. (2019). Bioactivity: Phenylpropanoids’ best kept secret. Curr. Opin. Biotechnol..

[29] Walker A.R., Lee E., Bogs J., McDavid D.A.J., Thomas M.R., Robinson S.P. (2007). White grapes arose through the mutation of two similar and adjacent regulatory genes. Plant J..

[30] Takos A.M., Jaffe F.W., Jacob S.R., Bogs J., Robinson S.P., Walker A.R. (2006). Light-induced expression of a MYB gene regulates anthocyanin biosynthesis in red apples. Plant Physiol..

[31] Ring L., Yeh S.-Y., Hücherig S., Hoffmann T., Blanco-Portales R., Fouche M., Villatoro C., Denoyes B., Monfort A., Caballero J.L. (2013). Metabolic Interaction between Anthocyanin and Lignin Biosynthesis Is Associated with Peroxidase FaPRX27 in Strawberry Fruit. Plant Physiol..

[32] Shaipulah N.F., Muhlemann J.K., Woodworth B.D., Van Moerkercke A., Verdonk J.C., Ramirez A.A., Haring M.A., Dudareva N., Schuurink R.C. (2016). CCoAOMT Down-Regulation Activates Anthocyanin Biosynthesis in Petunia. Plant Physiol..

[33] Song J.L., Wang Z.Y., Wang Y.H., Du J., Wang C.Y., Zhang X.Q., Chen S., Huang X.L., Xie X.M., Zhong T.X. (2022). Overexpression of Pennisetum purpureum CCoAOMT Contributes to Lignin Deposition and Drought Tolerance by Promoting the Accumulation of Flavonoids in Transgenic Tobacco. Front. Plant Sci..

[34] Yin R., Messner B., Faus-Kessler T., Hoffmann T., Schwab W., Hajirezaei M.-R., von Saint Paul V., Heller W., Schäffner A.R. (2012). Feedback inhibition of the general phenylpropanoid and flavonol biosynthetic pathways upon a compromised flavonol-3-O-glycosylation. J. Exp. Bot..

[35] Lai Z., Wang J., Fu Y., Wang M., Ma H., Peng S., Chang F. (2024). Revealing the role of CCoAOMT1: Fine-tuning bHLH transcription factors for optimal anther development. Sci. China Life Sci..

