# Supplementary material for: Integrated Transcriptomics and Metabolomics Analysis Reveals IbCCoAOMT7 Negatively Regulating Anthocyanin Accumulation in Sweetpotato Storage Roots
Source: Biology (Basel). 2026 Jul 8;15(14):1102. doi: 10.3390/biology15141102 (PMC13404952; doi:10.3390/biology15141102)
Supplement: Supplementary file 1 [file biology-15-01102-s001.zip › Figure S1.pdf]

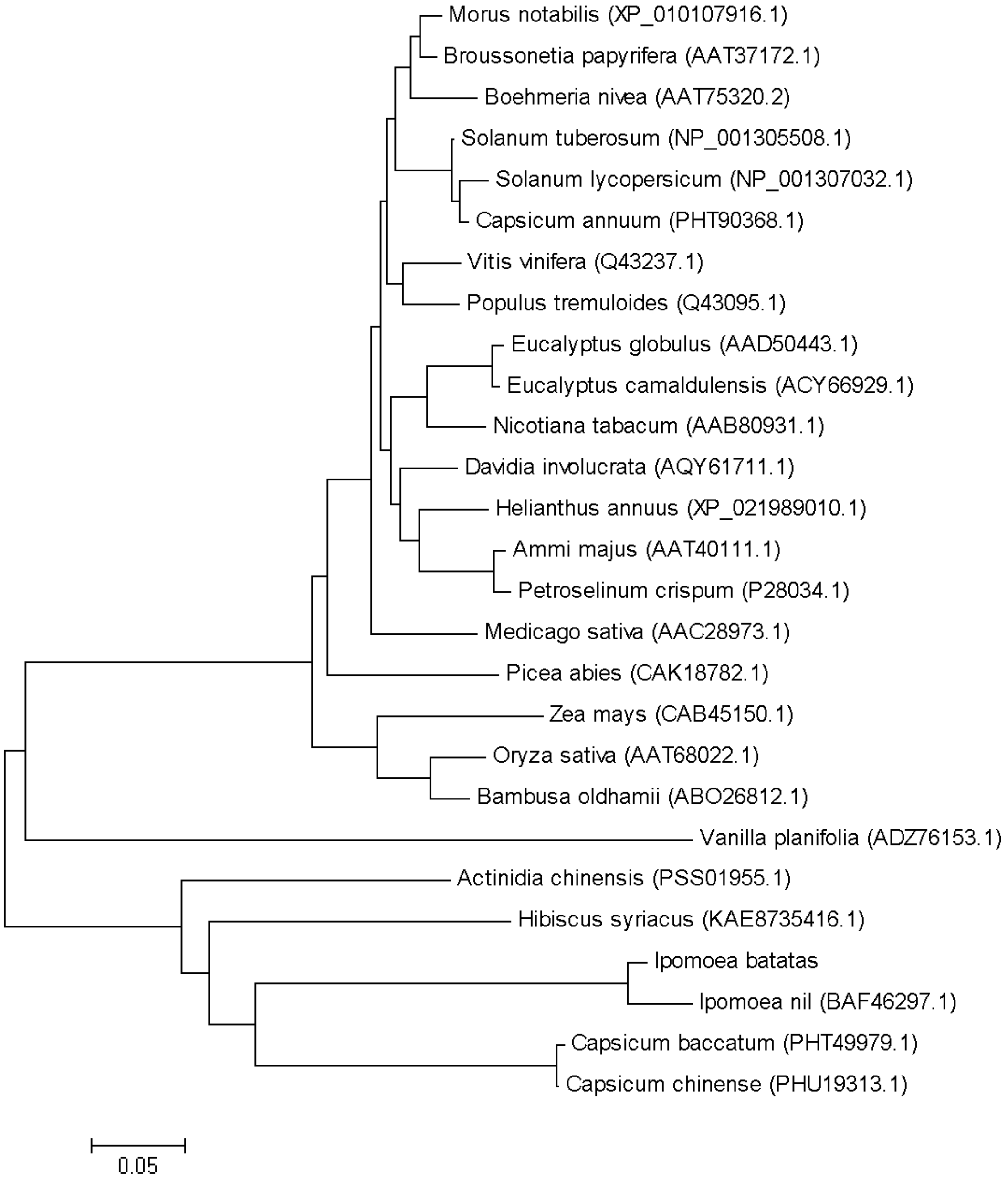

Figure S1 Phylogenetic tree of IbCCoAOMT protein from sweetpotato and CCoAOMT proteins from other species

Ipomoea batatas: IbCCoAOMT1; *Ipomoea nil*: BAF46297.1; *Zea mays*: CAB45150.1; *Oryza sativa*: AAT68022.1; *Eucalyptus globulus*: AAD50443.1; *Nicotiana tabacum*: AAB80931.1; *Picea abies*: CAK18782.1; *Vanilla planifolia*: ADZ76153.1; *Bambusa oldhamii*: ABO26812.1; *Boehmeria nivea*: AAT75320.2; *Medicago sativa*: AAC28973.1; *Vitis vinifera*: Q43237.1; *Ammi majus*: AAT40111.1; *Petroselinum crispum*: P28034.1; *Davidia involucrate*: AQY61711.1; *Helianthus annuus*: XP_021989010.1; *Morus notabilis*: XP_010107916.1; *Solanum tuberosum*: NP_001305508.1; *Broussonetia papyrifera*: AAT37172.1; *Populus tremuloides*: Q43095.1; *Eucalyptus camaldulensis*: ACY66929.1; *Solanum lycopersicum*: NP_001307032.1; *Capsicum annuum*: PHT90368.1; *Capsicum baccatum*: PHT49979.1; *Capsicum chinense*: PHU19313.1; *Actinidia chinensis*: PSS01955.1; *Hibiscus syriacus*: KAE8735416.1
