# Supplementary material for: Integrated Transcriptomics and Metabolomics Analysis Reveals IbCCoAOMT7 Negatively Regulating Anthocyanin Accumulation in Sweetpotato Storage Roots
Source: Biology (Basel). 2026 Jul 8;15(14):1102. doi: 10.3390/biology15141102 (PMC13404952; doi:10.3390/biology15141102)
Supplement: Supplementary file 1 [file biology-15-01102-s001.zip › Figure S2.pdf]

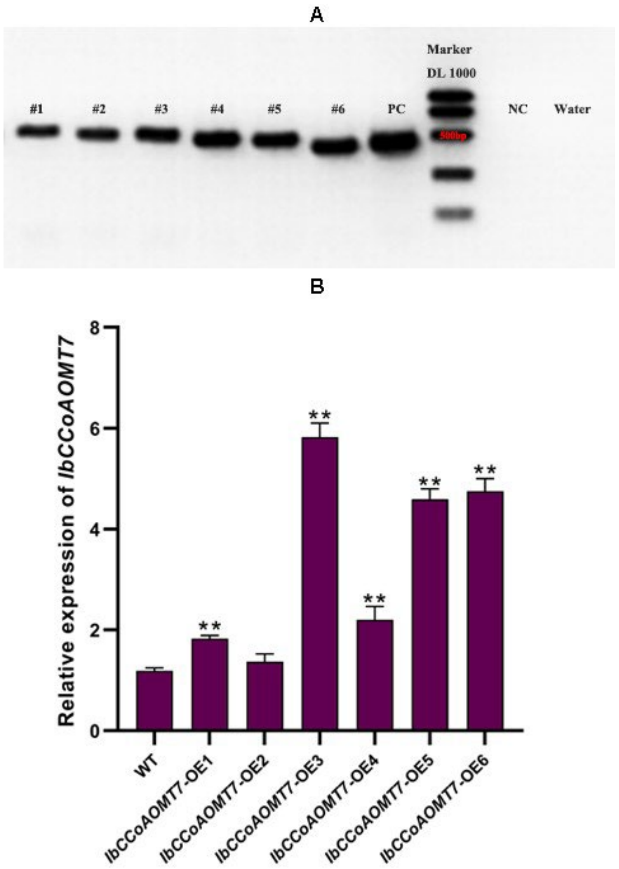

Figure S2 Comprehensive molecular validation of the generated IbCCoAOMT7 transgenic lines.

A. Genomic DNA PCR amplification utilized to confirm the genomic integration of the transgene within the XZ8 background. Specific lines denote independent transgenic events evaluated in parallel with a positive control labeled PC and a negative control labeled NC. B. Relative expression profiles of *IbCCoAOMT7* across the wild type and the respective overexpressing lines quantified via real time quantitative PCR. Error bars indicate the standard error derived from three independent biological samples. Asterisks signify highly significant differences relative to the wild type where the *p*-value＜0.05 according to the Student’t test.
